# Increased urinary Angiotensinogen/Creatinine (AGT/Cr) ratio may be associated with reduced renal function in autosomal dominant polycystic kidney disease patients

**DOI:** 10.1186/s12882-015-0075-8

**Published:** 2015-06-20

**Authors:** Hayne Cho Park, Ah-Young Kang, Joon Young Jang, Hyunsuk Kim, Miyeun Han, Kook-Hwan Oh, Seung Hyup Kim, Jung Woo Noh, Hae Il Cheong, Young-Hwan Hwang, Curie Ahn

**Affiliations:** Department of Internal Medicine, Armed Forces Capital Hospital, Seongnam, South Korea; Research Coordination Center for Rare Diseases, Seoul National University Hospital, Seoul, South Korea; Transplantation Research Institute, Seoul National University, Seoul, South Korea; Division of Nephrology, Department of Internal Medicine, Seoul National University Hospital, 101 Daehak-Ro Jongno-Gu, Seoul, 110-744 South Korea; Department of Radiology, Seoul National University Hospital, Seoul, South Korea; Department of Internal Medicine, Hallym University College of Medicine, Seoul, South Korea; Department of Pediatrics, Seoul National University Children’s Hospital, Seoul, South Korea; Department of Internal Medicine, Eulji University College of Medicine, Seoul, South Korea

**Keywords:** Angiotensinogen, Autosomal dominant polycystic kidney disease, Biomarkers, Renal function, Renin-angiotensin system

## Abstract

**Background:**

Autosomal dominant polycystic kidney disease (ADPKD) is one of the most common hereditary kidney diseases that frequently result in renal failure. In this cross-sectional observational cohort study, we evaluated urinary angiotensinogen (AGT) as a potential biomarker to assess renal function in ADPKD.

**Methods:**

Urinary AGT was measured in 233 ADPKD patients and its association with estimated glomerular filtration rate (eGFR) and height-adjusted total kidney volume (htTKV) were evaluated. The localization of AGT and other renin-angiotensin system (RAS)-related molecules were identified using immunohistochemistry in human ADPKD tissues.

**Results:**

Baseline urinary AGT/Cr was negatively correlated with CKD-EPI eGFR (*r*^*2*^ *=* 0.162, *P* < 0.001) and positively correlated with htTKV (*r*^*2*^ = 0.107, *P* < 0.001). Both urinary AGT/Cr and plasma renin activity levels were significantly elevated in hypertensive ADPKD patients. Among hypertensive subjects, urinary AGT/Cr was significantly increased in the advanced CKD stages (III-V) compared to early CKD stages (I-II) (28.6 ± 60.3 vs. 93.2 ± 139.3 μg/g, *P* < 0.001). Immunohistochemical study showed strong expression of AGT along the cyst-lining epithelial cells as well as the nearby compressed tubular epithelial cells.

**Conclusions:**

Our results suggested that urinary AGT/Cr may be a valuable biomarker for renal damage in ADPKD since intrarenal ischemic insults induced by cyst growth and subsequent intrarenal RAS activation may play a potential role in the development of hypertension and renal dysfunction in ADPKD.

**Electronic supplementary material:**

The online version of this article (doi:10.1186/s12882-015-0075-8) contains supplementary material, which is available to authorized users.

## Background

Autosomal dominant polycystic kidney disease (ADPKD) is one of the most common hereditary kidney diseases with the prevalence of 1 per 1000-4000 [1–3]. Renal failure is one of the most serious complications in ADPKD. Since the renal function starts to decline only after renal parenchyma has been substituted by abundant cysts and fibrotic tissues, there has been a growing need for discovering novel biomarkers that better reflect the degree of renal damage before renal function declines. Several biomarkers for renal dysfunction in ADPKD have been discovered and evaluated [4–9]. We also observed both urinary N-acetyl-β-D-glucosaminidase/creatinine (NAG/Cr) and β2-microglobulin/creatinine (β2MG/Cr) were associated with renal dysfunction in ADPKD [10]. However, previous studies failed to demonstrate the relationship between biomarker and underlying renal pathophysiology.

ADPKD patients develop hypertension earlier than the essential hypertensive patients and it is also well known that early-onset hypertension is the major predictor of renal outcome in ADPKD [11]. Intrarenal renin-angiotensin system (RAS) has been suggested as the main mechanism in the development of hypertension in ADPKD since cyst development and growth activate and accelerate intrarenal RAS far earlier than renal fibrosis and renal dysfunction. Urinary angiotensinogen (AGT) has been suggested as a biomarker reflecting the intrarenal RAS activity in hypertensive patients [12]. Moreover, urinary AGT levels were well correlated with renal function and the degree of albuminuria in a wide range of chronic kidney disease population [13–16]. Since RAS activation is closely linked to early-onset hypertension and subsequent renal damage, this study investigated 1) the association of urinary AGT/Cr to estimated glomerular filtration rate (eGFR) and height-adjusted TKV (htTKV), 2) its relationship with hypertensive status, and 3) tissue expression of AGT and other intrarenal RAS-related peptides in ADPKD human kidneys.

## Methods

### Study population

From January 2011 to February 2012, a total of 304 ADPKD patients above 18 years regularly visiting our ADPKD clinic were enrolled in the study. ADPKD was clinically diagnosed according to the Unified Criteria for Ultrasonographic Diagnosis of ADPKD proposed by Pei et al [17]. Among them, 71 patients were excluded from the analysis due to following reasons: 34 patients older than 60 years old, 5 patients on hemodialysis, 4 patients who received kidney transplantation, 12 patients with cancer, 8 patients currently on immunosuppressive therapy for the various reasons, 1 patient with previous unilateral nephrectomy, 2 patients who did not take computed tomography (CT) exam, 4 patients with chronic viral hepatitis, and 1 pregnant patient. As a result, a total of 233 patients were included in an analysis.

### Data collection

Upon enrollment, the demographic information and laboratory parameters were investigated. Systolic and diastolic blood pressures were measured at every visit and the number and types of anti-hypertensive medications were also reviewed. Upon enrollment, blood samples were withdrawn and serum Cr was measured by Jaffe method. The eGFR was calculated using chronic kidney disease epidemiology (CKD-EPI) equation [18, 19]. The chronic kidney disease (CKD) stage was defined according to the eGFR estimated by CKD-EPI equation as follows: stage I (>90 mL/min/1.73 m^2^), stage II (60-90 mL/min/1.73 m^2^), stage IIIA (45-60 mL/min/1.73 m^2^), stage IIIB (30-45 mL/min/1.73 m^2^), stage IV (15-30 mL/min/1.73 m^2^), and stage V (<15 mL/min/1.73 m^2^) [20]. To evaluate systemic RAS activity, plasma renin activity (PRA) and basal aldosterone were measured on resting state. Albumin and Cr were measured from random urine samples.

### Measurement of TKV

Of 233 patients, 200 patients underwent CT within 1 year from their enrollment time points. Thirty-three patients were excluded from the analysis because they underwent CT scans apart from urinary biomarker measurement (>1 year). Among them, 189 patients had available height information to calculate htTKV (mL/m) [21]. We have calculated TKV from 3- to 5-mm thickness three-dimensional contrast-enhanced CT of the kidney and bladder using a multi-detector CT scanner (Somatom Sensation 16, SIEMENS; Light speed Ultra 8, GE; Brilliance CT 64, Philips; Somatom Definition, SIEMENS). The CT examination was performed either with contrast agent or without depending on patient’s renal function. In order to acquire contrast images, we administered contrast material right after pre-contrast image acquisition and acquire post-contrast images about 17-20 s later when the Hounsfield Unit (HU) at the descending aorta reaches 100HU. The htTKV was defined as the sum of the left and right renal volumes divided by height of the patient.

### Measurement of urinary AGT

Urine samples were separately collected for the measurement of urinary AGT, mixed with 1 mL of 10 mM Tris buffer, centrifuged at 3000 g for 10 min, and stored at -80 °C until measurement. Urinary AGT was measured by commercial sandwich enzyme-linked immunosorbent assay (ELISA) (Immuno-Biological Laboratories, Co., Ltd., Gunma, Japan). Urine samples were diluted 10 times before the measurement. To evaluate whether urinary AGT is a better biomarker for renal function in ADPKD, we also measured urinary NAG and β2MG. The activity of urinary NAG was measured by a spectrophotometric assay under a 340-nm wavelength using a TBA 200 FR biochemical analyzer (Toshiba, Tokyo, Japan) [22]. The urinary excretion level of β2MG was measured using a radioimmunoassay kit (Beckman Coulter, Prague, Czech Republic) [23]. To compensate for the production of concentrated or dilute urine samples, the urinary biomarker levels were expressed based on urinary Cr content.

### Immunohistochemistry

One normal kidney specimen and two polycystic kidney specimens were collected from radical nephrectomized kidneys (Table 1). Each kidney specimen was fixed in 4 % paraformaldehyde (PFA) for 24 h and paraffin embedded. The 4-μm sections were prepared for immunohistochemistry as described before [24]. Antigen retrieval was performed with Tris-EDTA buffer, pH9.0, at 100 °C for 15 min. The slides were rinsed three times with PBS, followed by the addition of 3 % H_2_O_2_ for 10 min at room temperature to block endogenous peroxidase. The slides were rinsed three times with PBS and then blocker with universal blocking buffer. The primary antibodies were added at the dilution indicated in Table 2. Slides were incubated either for 1 h at room temperature or overnight at 4 °C, followed by the addition of the secondary antibodies. Sections were stained with DAB substrate and counterstained with hematoxylin, and viewed under a Leica microscope.

**Table 1:** Clinical characteristics of the nephrectomized patients

	Normal control	Case I	Case II
	(Normotensive)	(PKD-CKD)	(PKD-ESRD)
Gender/Age (yr)	M / 38	F / 48	M / 61
Diagnosis	Renal cell carcinoma	Renal cell carcinoma, ADPKD	ADPKD
Creatinine (mg/dL)	1.1	0.98	5.4
Hypertension duration (yr)	-	14	12
Hypertension medication	0	Valsartan 160 mg, Tenormin 12.5 mg	Candesartan 8 mg, Amlodipine 10 mg
Systolic/diastolic blood pressure (mmHg)	120 / 70	134 / 91	137 / 85

ADPKD, autosomal dominant polycystic kidney disease; CKD, chronic kidney disease; ESRD, end-stage renal disease

**Table 2:** Primary antibodies used in the immunohistochemistry

Target Molecule	Immunogen	Host	Type	Source	Dilution
AGT	Human AGT	Mouse	Monoclonal	R&D Systems	1:600
AngII	Human angiotensin II	Rabbit	Polyclonal affinity purified	Novus Biologicals	1:100
Ang-(1-7)	Rat angiotensin (1-7)	Rabbit	Polyclonal affinity purified	Alomone Labs	1:100
ACE	Human ACE	Mouse	Monoclonal	Abcam	1:50
ACE2	NS0-derived rhACE-2 ectodomain	Goat	Polyclonal affinity purified	R&D Systems	1:10
Chymase	Human chymase (skin)	Mouse	Polyclonal affinity purified	Chemicon International	1:500

*ACE* angiotensin converting enzyme, *ACE2* angiotensin converting enzyme 2, *AngII* angiotensin II, *Ang-(1-7)* angiotensin (1-7), *AGT* angiotensinogen

### Statistical analyses

Statistical analyses were performed using SPSS, version 19.0 (SPSS Inc., www.spss.com). The variables that did not follow normal distribution were log-transformed before analysis. To investigate whether urinary biomarkers were correlated with either eGFR or htTKV, linear regression analyses were performed. To adjust for potential confounders, multiple regression analysis was performed. We also performed independent *t*-test and the analysis of variance (ANOVA) test to compare continuous variables between groups. The *P*-value < 0.05 was considered statistically significant.

### Ethics statement

This study was approved by the Institutional Review Board of Seoul National University Hospital (H-0901-046-269). The procedures for the use of the human kidney specimens were approved by Seoul National University Hospital Institutional Review Board (H-0701-033-195). All participants provided written informed consent before the study in accordance to the Declaration of Helsinki.

## Results

### Clinical characteristics of participants

A total of 233 ADPKD patients were selected for this study. Baseline clinical characteristics are summarized in Table 3. The mean age was 43.3 ± 9.7 years and had slight female predominance (54.1 %). The majority of them were either diagnosed with hypertension or on blood pressure lowering therapy (n = 217, 93.1 %), and 153 patients were taking angiotensin converting enzyme inhibitor (ACEi) and/or angiotensin receptor blocker (ARB). Mean serum Cr and eGFR were 1.1 ± 0.5 mg/dL and 80.5 ± 27.1 mL/min/1.73 m^2^, respectively. The median value of urinary AGT/Cr was 13.7 (Interquartile range, IQR 7.5 – 35.1) μg/g. The CT scan was taken in 189 subjects. Measured htTKV at baseline was 705 mL/m (Min. 119, Max. 3436) and the mean time gap between urinary biomarker measurement and CT scan was 4.7 ± 4.0 months.

**Table 3:** Baseline clinical characteristics of participants

Parameters	Patients (N = 233)	Patients with htTKV (N = 189)	Patients without htTKV (N = 44)	P-value
Age (yr)	43.3 ± 9.7	43.8 ± 9.3	41.2 ± 11.3	0.198
Female	126 (54.1 %)	99 (52.4 %)	27 (61.4 %)	0.316
Age at diagnosis (yr)	34.7 ± 9.2	35.2 ± 9.2	32.2 ± 8.9	0.114
Hypertension	217 (93.1 %)	147 (77.8 %)	29 (65.9 %)	0.119
Duration of hypertension (yr)	8.6 ± 6.0	8.6 ± 5.9	8.6 ± 6.6	0.67
Systolic BP (mmHg)	129.8 ± 13.5	130.0 ± 13.8	129.0 ± 12.6	0.404
Diastolic BP (mmHg)	80.0 ± 9.9	80.5 ± 10.0	78.1 ± 9.4	0.049
Number of BP medication	1 (Min. 0, Max. 7)	1 (Min. 0, Max. 7)	1 (Min. 0, Max. 4)	0.686
ARB or ACEi medication (%)	153 (65.7 %)	122 (64.6 %)	30 (68.2 %)	0.825
HtTKV (mL/m)	-	705 (Min 119, Max. 3436)	-	-
Time gap between enrollment and CT volumetry (mo)	-	4.7 ± 4.0	-	-
Hemoglobin (g/dL)	13.4 ± 1.5	13.4 ± 1.6	13.3 ± 1.3	0.592
Creatinine (mg/dL)	1.1 ± 0.5	1.2 ± 0.5	0.9 ± 0.3	0.003
CKD-EPI eGFR (mL/min/1.73 m^2^)	80.5 ± 27.1	77.9 ± 27.1	91.7 ± 24.9	0.002
Random urine microalbumin-to-creatinine ratio (mg/g)	21.0 (9.0–64.5)	23.0 (11.0–66.5)	15.0 (6.0–43.0)	0.076
PRA (ng/mL/hr)	3.6 (1.4–8.2)	3.9 (1.4–9.0)	3.0 (0.9–5.3)	0.067
Plasma aldosterone (ng/dL)	15.8 (11.7–21.6)	14.9 (11.4–21.1)	18.3 (12.3–23.7)	0.232
Urinary AGT/Cr (μg/g)	13.7 (7.5–35.1)	14.9 (7.8–36.0)	13.1 (6.2–30.8)	0.289
Urinary NAG/Cr (IU/g)	3.9 (2.7–5.9)	4.0 (2.9–5.9)	3.5 (2.3–6.1)	0.206
Urinary β2MG/Cr (μg/g)	310.6 (214.3–560.0)	305.8 (215.9–552.9)	329.1 (206.3–654.6)	0.871

Data are expressed as numbers (percentages), mean ± SD, or median (Interquartile range). HtTKV was measured in 189 patients with available height and CT volumetry. *ACEi* angiotensin converting enzyme inhibitor, *AGT* angiotensinogen, *ARB* angiotensin receptor blocker, *β2MG* β2-microglobulin, *BP* blood pressure, *CKD-EPI eGFR* chronic kidney disease epidemiology estimated glomerular filtration rate, *HtTKV* height-adjusted total kidney volume, *PRA* plasma renin activity, *NAG* N-acetyl-β-D-glucosaminidase. Urinary concentrations of biomarkers were log-transformed to fulfill the requirement of normal distribution of residuals

### Urinary AGT was correlated with eGFR and htTKV

**Fig. 1:**
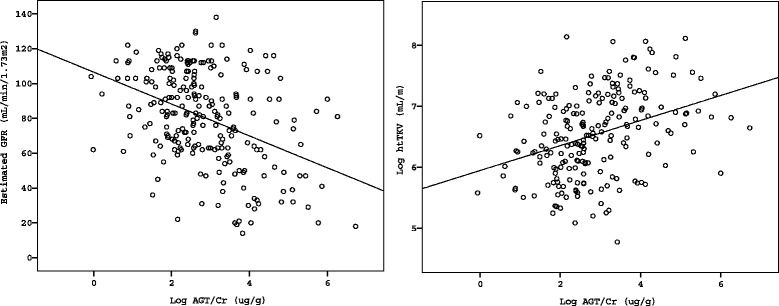
Linear Regression Analysis Between Urinary Angiotensinogen/Creatinine Ratio, eGFR, and htTKV. Urinary angiotensinogen/creatinine ration (AGT/Cr) showed negative correlation with eGFR (*r* ^*2*^ = 0.162, *P* < 0.001) and positive correlation with htTKV (*r* ^*2*^ = 0.107, *P* < 0.001)

**Fig. 2:**
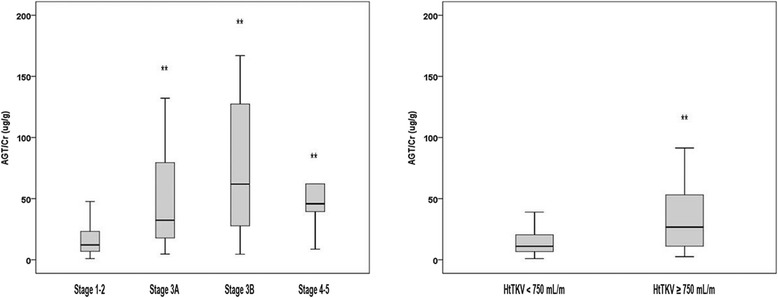
Urinary AGT/Cr According to CKD Stages and htTKV. The urinary AGT/Cr value was significantly increased as CKD stages progressed (CKD stage 1-2 (n = 186), 27.8 ± 58.5 μg/g; CKD stage 3A (n = 22), 56.0 ± 61.1 μg/g; CKD stage 3B (n = 15), 89.0 ± 89.5 μg/g; CKD stage 4-5 (n = 9), 95.3 ± 108.9 μg/g). In addition, the subjects with larger htTKV (≥750 mL/m) demonstrated greater urinary AGT/Cr level (50.9 ± 76.7 vs. 23.5 ± 48.2 μg/g, *P* = 0.003). ** Each value showed statistically significant difference (*P* < 0.05) compared to the reference value (CKD stage I-II, HtTKV < 750 mL/m). AGT, angiotensinogen; CKD, chronic kidney disease; Cr, creatinine; htTKV, height-adjusted total kidney volume

To evaluate the association between each urinary biomarker and renal functional and structural markers, a linear regression analyses were performed. Urinary AGT, NAG, and β2MG were compared with eGFR, serum Cr, and htTKV. Urinary AGT/Cr was negatively correlated with eGFR (*r*^*2*^ = 0.162, *P* < 0.001)(Fig. 1). When compared with urinary NAG/Cr and β2MG/Cr, urinary AGT/Cr showed the best correlation with eGFR demonstrating the greatest Pearson’s correlation coefficient (*r*^*2*^ = 0.162 vs. 0.111 vs. 0.138, *P* < 0.001) (Additional file 1: Figure S1). Furthermore, when we performed ANOVA among CKD stage groups, the mean values of urinary AGT/Cr increased according to CKD stages (CKD stage I-II (n = 186), 27.8 ± 58.5 μg/g; CKD stage IIIA (n = 22), 56.0 ± 61.1 μg/g; CKD stage IIIB (n = 15), 89.0 ± 89.5 μg/g; CKD stage IV-V (n = 9), 95.3 ± 108.9 μg/g) (Fig. 2).

Among 189 patients with available htTKV measurement, urinary AGT/Cr also demonstrated a better correlation with htTKV (*r*^*2*^ = 0.107, *P* < 0.001) than urinary NAG/Cr and β2MG/Cr (*r*^*2*^ = 0.089 and *r*^*2*^ = 0.08, *P* < 0.001) (Additional file 1: Figure S1). The patients with larger htTKV > 750 mL/m (n = 90) showed greater urinary AGT/Cr (50.9 ± 76.7 μg/g) compared with those with smaller kidneys < 750 mL/m (23.5 ± 48.2 μg/g, *P* = 0.003) (Fig. 2). These results indicated that urinary AGT/Cr shows a good correlation with both concurrent eGFR and htTKV.

### Urinary AGT demonstrated better association with CKD stages compared to PRA in hypertensive ADPKD patients

**Fig. 3:**
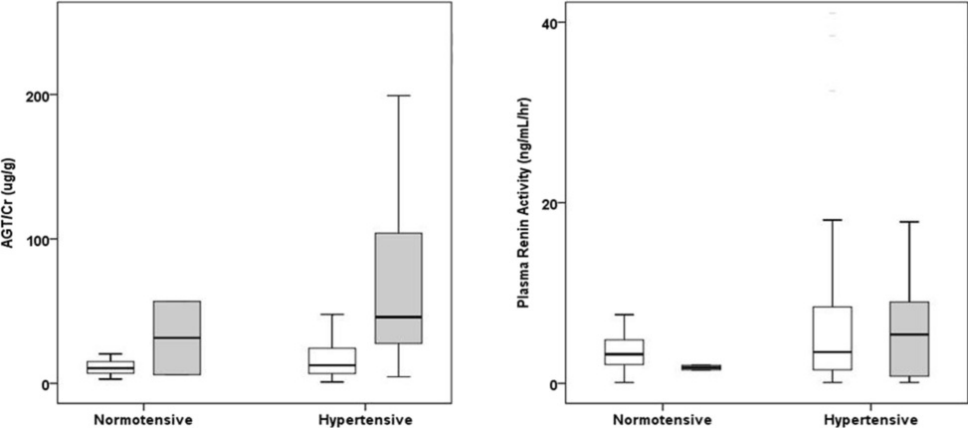
Urinary AGT/Cr and PRA Levels According to CKD Stages and Hypertension. As a marker of intrarenal RAS activation, we measured urinary AGT/Cr and compared with PRA level, a marker of systemic RAS activation. Both urinary AGT/Cr and PRA were elevated in hypertensive subjects compared to normotensive subjects (*P* > 0.05). Among hypertensive subjects, urinary AGT/Cr was significantly increased in the advanced CKD stages (III-V, gray bar) compared to early CKD stages (I-II, white bar)(28.6 ± 60.3 vs. 93.2 ± 139.3 μg/g, *P* < 0.001). In contrast, PRA levels were not statistically different between CKD stage groups. AGT, angiotensinogen; CKD, chronic kidney disease; Cr, creatinine; PRA, plasma renin activity

Since RAS activation is a major cause of hypertension in ADPKD patients, urinary AGT/Cr and PRA levels were compared among normotensive and hypertensive groups. As shown in Fig. 3, both urinary AGT/Cr and PRA were tended to be higher in hypertensive subjects compared to normotensive subjects (*P* > 0.05). Among hypertensive subjects, urinary AGT/Cr was significantly increased in the advanced CKD stages (III-V) compared to early CKD stages (I-II) (28.6 ± 60.3 vs. 93.2 ± 139.3 μg/g, *P* < 0.001). This remained statistically significant when we compared urinary AGT/Cr among the subgroup population who were not taking RAS blocker medications (29.9 ± 54.0 vs. 106.7 ± 217.7 μg/g, *P* = 0.009, data not shown). On the other hand, PRA could not differentiate those with decreased renal function from those with normal renal function (7.5 ± 8.7 vs. 7.4 ± 10.2 ng/mL/hr, *P* = 0.928). In concordance to previous studies, our results suggest that local RAS (represented by urinary AGT/Cr) as well as systemic RAS (represented by PRA) are activated in hypertensive patients. However, urinary AGT/Cr showed better association with CKD stages among hypertensive subjects compared to PRA. Meanwhile, RAS blockers, ACEi and/or ARB, did not affect either urinary AGT/Cr levels or PRA/aldosterone levels (Additional file 1: Figure S2).

### Urinary AGT was not an independent risk factor for reduced eGFR

To evaluate clinical variables associated with eGFR, we performed a linear regression analysis. Age, gender, and hypertension were evaluated as demographic variables. The eGFR at the initial hospital visit, plasma hemoglobin, serum uric acid, htTKV, random urine albumin to Cr ratio, urinary AGT/Cr were included as laboratory parameters. In univariate analysis, higher urinary AGT/Cr was associated with reduced eGFR as well as old age, hypertension, low initial eGFR, low plasma hemoglobin, high serum uric acid, large htTKV, and microalbuminuria (Table 4). Since the association between urinary AGT/Cr and eGFR may be confounded by other clinical variables, multivariate regression analyses were performed as the next step. The stepwise selection method was used to define independent factors. Age, hypertension, initial eGFR, plasma hemoglobin, serum uric acid, htTKV, random urine albumin to Cr ratio, urinary AGT/Cr were included in the final model. Old age, low initial eGFR, low plasma hemoglobin, high serum uric acid, and large htTKV were the independent factors associated with reduced eGFR. However, urinary AGT/Cr was not an independent factor associated with reduced eGFR.

**Table 4:** Linear regression analysis for risk factors for decreased estimated glomerular filtration rate

Parameters	Univariable		Multivariable	
	B (95 % CI)	P value	B (95 % CI)	P value
Age (yr)	-1.14 (-1.3 to -0.9)	<0.001	-0.5 (-0.66 to -0.33)	<0.001
Gender	1.97 (-4.4 to 8.3)	0.54		
Hypertension	-21.1 (-28.1 to -14.1)	<0.001		
Initial eGFR (mL/min/1.73 m^2^)	0.67 (0.6 to 0.7)	<0.001	0.3 (0.22 to 0.38)	<0.001
Hemoglobin (g/dL)	6.86 (5.1 to 8.6)	<0.001	2.91 (1.61 to 4.21)	<0.001
Uric acid (mg/dL)	-8.84 (-10.7 to -6.95)	<0.001	-4.22 (-5.66 to -2.78)	<0.001
HtTKV (mL/m)	-18.8 (-22.6 to -15.0)	<0.001	-5.6 (-8.57 to -2.63)	<0.001
Albumin/Cr (mg/g)	-11.2 (-13.3 to -9.12)	<0.001	-3.31 (-4.99 to -1.64)	<0.001
Urinary AGT/Cr (μg/g)	-9.5 (-11.8 to -7.19)	<0.001	-1.63 (-3.74 to 0.48)	0.1

In multivariate analysis, data were adjusted for age, hypertension, initial eGFR, plasma hemoglobin, serum uric acid, htTKV, random urine albumin-to-creatinine ratio, and urinary AGT/Cr by stepwise selection method. HtTKV, albumin/Cr, and AGT/Cr were log-transformed to fulfill the requirement of normal distribution of residuals. *AGT* angiotensinogen, *Cr* creatinine, *eGFR* estimated glomerular filtration rate, *htTKV* height-adjusted total kidney volume

### AGT was overexpressed in cyst-lining epithelial cells and proximal tubules of ADPKD compared to normal kidneys

**Fig. 4:**
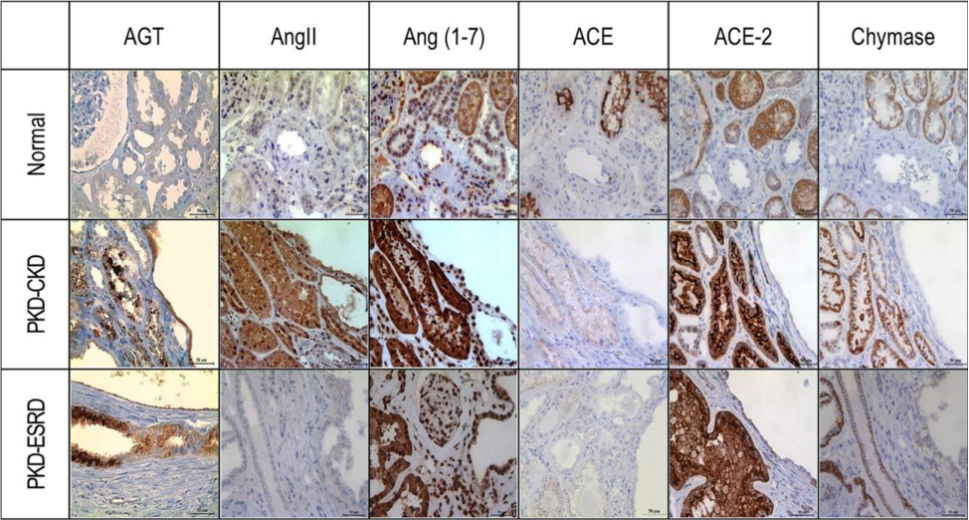
Immunohistochemistry of Intrarenal RAS Components in Polycystic Kidneys. Immunohistochemistry was performed to evaluate the expression levels of intrarenal RAS components in the polycystic kidneys (PKD-CKD and PKD-ESRD) compared to normal control kidneys. AGT was highly expressed in the proximal tubules and cyst-lining epithelial cells in polycystic kidneys whereas normal kidney did not express AGT in either glomeruli or tubules. Other intrarenal RAS components (AngII, Ang-(1-7), ACE2, and chymase) were highly expressed in polycystic kidneys compared to the normal kidney. However, the expression level of ACE was lower in the polycystic kidneys compared to the normal control. Magnification x400. ACE, angiotensin converting enzyme; ACE2, angiotensin converting enzyme 2; AGT, angiotensinogen; AngII, angiotensin II; Ang-(1-7), Angiotensin (1-7); CKD, chronic kidney disease; ESRD, end-stage renal disease; PKD, polycystic kidney disease; RAS, renin-angiotensin system

In order to investigate the origin of AGT expression in polycystic kidneys, immunohistochemistry was performed using normal and polycystic kidney tissues. In the normal kidney, AGT was not expressed in any of proximal tubules, glomeruli, or vessels. On the other hand, in case I (PKD-CKD), AGT was strongly expressed in proximal tubules and cyst-lining epithelial cells (Fig. 4). Of note, the staining intensity of AGT was greater in the proximal tubules compressed by nearby cysts. In case II, PKD-end-stage renal disease (ESRD), AGT was also expressed in the proximal tubules; however, its intensity was slightly reduced than that of case I. Nevertheless, strong expression of AGT was also noted at cyst-lining epithelial cells in case II.

### Other intrarenal RAS components were highly expressed in ADPKD

Expression levels of other intrarenal RAS components such as AngII, Ang-(1-7), ACE, ACE2, and chymase were investigated using immunohistochemical staining (Fig. 4, Table 5). Immunohistochemitry results of case I demonstrated that all other intrarenal RAS components including AngII, Ang-(1-7), ACE2 and chymase but ACE expression were augmented in the polycystic kidneys compared to the normal kidney. The AngII expression was moderately increased in both proximal and distal tubules. The Ang-(1-7) expression was markedly increased in proximal and distal tubules and glomeruli. The ACE2 expression was markedly increased in the proximal tubular cells of polycystic kidneys. However, the ACE2 expression level in the distal tubules was similar to that of normal kidney. Of note, the expression level of chymase, an alternative enzyme which converts AngI to AngII, was moderately increased in both proximal and distal tubules. Meanwhile, ACE expression levels were decreased in polycystic kidney tissue compared to the stain intensity in normal kidney tissue. Immunochemistry results of case II showed similar distribution of expression to case I, but cyst-lining epithelial cells were positively stained especially for Ang-(1-7). The staining pattern of AngII, ACE2 and chymase in polycystic kidney tissue was rather patchy with less staining intensity compared to those in case I.

**Table 5:** Tissue expression of intrarenal renin-angiotensin-aldosterone system components in polycystic kidneys compared to normal kidney

		AGT	AngII	Ang-(1-7)	ACE	ACE2	Chymase
Normal	PT	±	+	++	+++	++	+
	DT	-	+	+	-	++	+
Case I (ADPKD-CKD)	C	++(diffuse)	++	++	-	±	-
	PT	+++(diffuse)	++	+++	+	++++	+++
	DT	-	++	+++	-	++	++
Case II (ADPKD-ESRD)	C	++(patchy)	±	+++	-	+	+
	PT	+++(patchy)	±	+++	±	++++	+++
	DT	-	±	+++	-	++	++

Normal kidney was obtained from 38-year-old male who underwent nephrectomy for renal cell carcinoma. His sCr level was 1.1 mg/dL at the time of operation. The polycystic kidney tissues were obtained from two patients. One patient underwent nephrectomy for renal cell carcinoma and her sCr level was 0.98 mg/dL. The other kidney tissue was obtained from end-stage renal disease patient who was preparing transplantation. *ACE* angiotensin-converting enzyme, *ACE2* angiotensin-converting enzyme 2, *AGT* angiotensinogen, *AngII* angiotensin II, *Ang-(1-7)* angiotensin (1-7), *C* cyst-lining epithelial cells, *DT* distal tubules, *ADPKD-CKD* polycystic kidney disease – chronic kidney disease stage, *ADPKD-ESRD* polycystic kidney disease – end-stage renal disease stage, *PT* Proximal tubule

## Discussion

AGT is a 52- to 64- kD peptide molecule that has a limited glomerular permeability and tubular degradation. Because of these properties, urinary AGT has been studied as a marker for the intrarenal RAS activity among other RAS related proteins [25]. As a novel biomarker that reflects intrarenal RAS activity, urinary AGT has been reported to be associated with hypertension, proteinuria, and renal dysfunction [12, 13, 25]. It has recently been recognized as an important urinary biomarker for hypertension in ADPKD patients [26].

Our study is the largest study to demonstrate urinary AGT/Cr as a useful clinical parameter associated with renal dysfunction and underlying pathophysiology. In this study, urinary AGT/Cr demonstrated a better association with concurrent eGFR, htTKV, and hypertension compared to other biomarkers including urinary NAG/Cr and β2MG/Cr. In addition, our immunohistochemical staining results showed that most of RAS components including AGT were highly expressed in the polycystic kidney tissues compared to normal kidney. These results suggest that intrarenal RAS activation may play an important role in blood pressure elevation and renal dysfunction. Indeed, high blood pressure often manifests far earlier than cyst growth and replacement of renal parenchyma. It suggests that not only mechanical compression by cysts but also paracrine hormonal action will be crucial for developing hypertension in ADPKD.

In our study, urinary AGT/Cr was well correlated with concurrent eGFR and htTKV even in the early CKD stages. In the previous study, urinary AGT/Cr measured from healthy volunteers ranged from 5.0 to 30.0 μg/g (Additional file 1: Table S1) [27]. In our study, urinary AGT/Cr levels in ADPKD patients began to increase from as early as CKD stage I-II (27.8 ± 58.5 μg/g), showing higher value than those in hypertensive patients without RAS blocker usage [12]. It suggests that urinary AGT/Cr may reflect early stage renal damage well before renal functional (serum Cr or eGFR) and structural markers (htTKV) change. In addition, the fact that urinary AGT/Cr was higher in the patients with hypertension compared to normotensive patients clearly show that urinary AGT/Cr can be a meaningful biomarker that reflects underlying pathophysiology of ADPKD. However, in multivariate analysis, the association between urinary AGT/Cr and eGFR was disappeared after adjustment with other clinical variables including age, gender, hypertension, and initial eGFR. Like many other biomarkers for ADPKD [8], independent effect of urinary AGT/Cr seemed to be reduced by the impact of initial eGFR, which may pose strong impact on the subsequent eGFR. In addition, various confounding factors may affect urinary AGT/Cr levels such as medication, ACE gene polymorphisms and diet.

In this study, the use of ACE inhibitors and/or ARBs did not result in decreased urinary AGT/Cr. It is hard to directly compare this with previous studies because most of previous studies were performed in the prospective trial design. However, there are several possible explanations. First, the information about the duration of RAS blocker prescription was not collected and analyzed. This is important because intrarenal RAS activity seems to rebound sometime after using RAS blockers. Jang *et al.* clearly showed this in their previous study that urinary AGT/Cr was increased again after 3 months of RAS blocker medication [28]. Second, there is a possibility that inadequate amount of RAS blockers was used that could not suppress intrarenal AngII activity.

However, our immunohistochemical study suggests AGT may be produced and secreted vigorously in the early CKD stages. In concordance to previous study results, AGT was highly expressed in proximal tubular epithelial cells and cyst-lining cells of ADPKD [24]. Notably, the expression level of AGT was much stronger in case I (PKD-CKD, eGFR 69 mL/min/1.73 m^2^ with hypertension) than that in case II (PKD-ESRD, eGFR 11 mL/min/1.73 m^2^ with hypertension, pre-dialysis) suggesting that urinary AGT/Cr may be more useful in the early stage of renal disease. Previous studies support our results, clearly showing that all the intrarenal RAS components are highly expressed in the proximal tubules in the pathologic condition [24, 28, 29]. Proximal tubular cells can actively produce AngII and also secrete AGT into the urine [30]. Intraluminal AGT may be converted in the distal tubules to AngII, which may lead to induction of sodium channels and aldosterone production to elevate blood pressure [31].

Unravelling the mechanisms involving intrarenal RAS activation in ADPKD is beyond the scope of this study since this study aims to investigate the clinical usefulness of urinary AGT/Cr as a biomarker of renal insufficiency. However, it is worthy to note that the expression levels of the counterpart molecules, ACE2 and Ang-(1-7), were also elevated in the polycystic kidneys. Two possible explanations can be given. First, since the subjects were taking ARB to block the action of AngII, the feedback mechanism would increase the production of upstream molecules, AGT and AngII. Ang II is then further converted to Ang (1-7) directly by ACE. Another possible mechanism is that the dose of RAS blocker was insufficient to decrease intrarenal RAS activity hence the counterpart pathway was activated to compensate the deleterious effects of AngII. The functional effect of increased ACE2 and Ang-(1-7) should be demonstrated in the further study.

The expression of ACE was universally decreased in ADPKD tissue compared to normal control. Previous study suggested that AngII production in organ or tissue may rely more on chymase rather than ACE in pathologic conditions [32]. The other study also demonstrated that AngII activity in heart, aorta, and lung under the hypertensive condition was more dependent on chymase activity rather than ACE activity [33]. Therefore, in pathologic conditions, chymase activity may overtake the role of ACE in the production of AngII.

This study has several limitations. This is a single-center, cross-sectional study that included only Korean patients. Second, we failed to show independent association between urinary AGT/Cr and eGFR. Third, patients in advanced CKD stages were largely excluded. Lastly, TKV was measured using a modified ellipsoid method from CT images. The modified ellipsoid method was originally developed to measure kidney volumes from ultrasonographic images [34]. In addition, most previous studies measured TKV by computer-based volumetry using magnetic resonance imaging (MRI). Further validation studies are warranted to use this method in CT volumetry.

## Conclusions

We found that high urinary level of AGT/Cr was associated with lower eGFR, larger htTKV, and hypertension. Immunohistochemical results demonstrated the possible link between ischemic insult by cyst growth and subsequent activation of intrarenal RAS in the progression of ADPKD. Further long-term studies should reveal the usefulness of urinary AGT/Cr in the prediction of faster renal function deterioration.

## Additional file

Additional file 1: Figure S1. Association Between Urinary Biomarker Concentration, eGFR, serum Cr, and htTKV. All patients measured urinary angiotensinogen (AGT), N-acetyl- β-D-glucosaminidase (NAG), and β2-microglobulin (β2MG). Urinary biomarker concentration was expressed based on urinary Cr contents. The concentration of each urinary biomarker, serum Cr, and htTKV were log-transformed before analysis. Among them, 189 patients had available htTKV measurement within 1 year of enrollment. Among other biomarkers, urinary AGT/Cr showed a better association with eGFR (*r* ^*2*^ = 0.162, *P* < 0.001) and htTKV (*r* ^*2*^ = 0.107, *P* < 0.001). **Figure S2.** Urinary AGT/Cr, PRA, and Plasma Aldosterone Levels According to RAS Blocker Usage. Urinary AGT/Cr was not statistically different between patients with RAS blocker usage (n = 153, 39.5 ± 70.2 μg/g) and those without RAS blocker usage (n = 64, 47.9 ± 117.6 μg/g, *P* = 0.595). In addition, PRA and plasma aldosterone, markers of systemic RAS activation, did not differ between groups (*P* >0.05). **Table S1.** Comparison of Urinary AGT/Cr Levels among Different Studies.

## Abbreviations

ACE: Angiotensin converting enzyme
ACE2: Angiotensiin converting enzyme 2
ACEi: Angiotensin converting enzyme inhibitor
ADPKD: Autosomal dominant polycystic kidney disease
AGT: Angiotensinogen
AngII: Angiotensin II
Ang-(1-7): Angiotensin (1-7)
ANOVA: Analysis of variance
ARB: Angiotensin receptor blocker
β2MG: β2-microglobulin
CKD: Chronic kidney disease
CKD-EPI: Chronic kidney disease epidemiology
Cr: Creatinine
CT: Computed tomography
eGFR: Estimated glomerular filtration rate
ELISA: Enzyme-linked immunosorbent assay
ESRD: End-stage renal disease
htTKV: Height-adjusted total kidney volume
HU: Hounsfield Unit
MRI: Magnetic resonance imaging
NAG: N-acetyl-β-D-glucosaminidase
PFA: Paraformaldehyde
PRA: Plasma renin activity
RAS: Renin-angiotensin system
