# Supplementary material for: Increased urinary Angiotensinogen/Creatinine (AGT/Cr) ratio may be associated with reduced renal function in autosomal dominant polycystic kidney disease patients
Source: BMC Nephrol. 2015 Jun 20;16:86. doi: 10.1186/s12882-015-0075-8 (PMC4475321; doi:10.1186/s12882-015-0075-8)
Supplement: Additional file 1: Figure S1. — Association Between Urinary Biomarker Concentration, eGFR, serum Cr, and htTKV. All patients measured urinary angiotensinogen (AGT), N-acetyl- β-D-glucosaminidase (NAG), and β2-microglobulin (β2MG). Urinary biomarker concentration was expressed based on urinary Cr contents. The concentration of each urinary biomarker, serum Cr, and htTKV were log-transformed before analysis. Among them, 189 patients had available htTKV measurement within 1 year of enrollment. Among other biomarkers, urinary AGT/Cr showed a better association with eGFR (r 2 = 0.162, P < 0.001) and htTKV (r 2 = 0.107, P < 0.001). Figure S2. Urinary AGT/Cr, PRA, and Plasma Aldosterone Levels According to RAS Blocker Usage. Urinary AGT/Cr was not statistically different between patients with RAS blocker usage (n = 153, 39.5 ± 70.2 μg/g) and those without RAS blocker usage (n = 64, 47.9 ± 117.6 μg/g, P = 0.595). In addition, PRA and plasma aldosterone, markers of systemic RAS activation, did not differ between groups (P >0.05). Table S1. Comparison of Urinary AGT/Cr Levels among Different Studies. [file 12882_2015_75_MOESM1_ESM.doc]

**Supplementary Figure 1. Association Between Urinary Biomarker Concentration, eGFR, serum Cr, and htTKV.** All patients measured urinary angiotensinogen (AGT), N-acetyl- β-D-glucosaminidase (NAG), and β2-microglobulin (β2MG). Urinary biomarker concentration was expressed based on urinary Cr contents. The concentration of each urinary biomarker, serum Cr, and htTKV were log-transformed before analysis. Among them, 189 patients had available htTKV measurement within 1 year of enrollment. Among other biomarkers, urinary AGT/Cr showed a better association with eGFR (*r^2^* = 0.162, *P* < 0.001) and htTKV (*r^2^* = 0.107, *P* < 0.001).


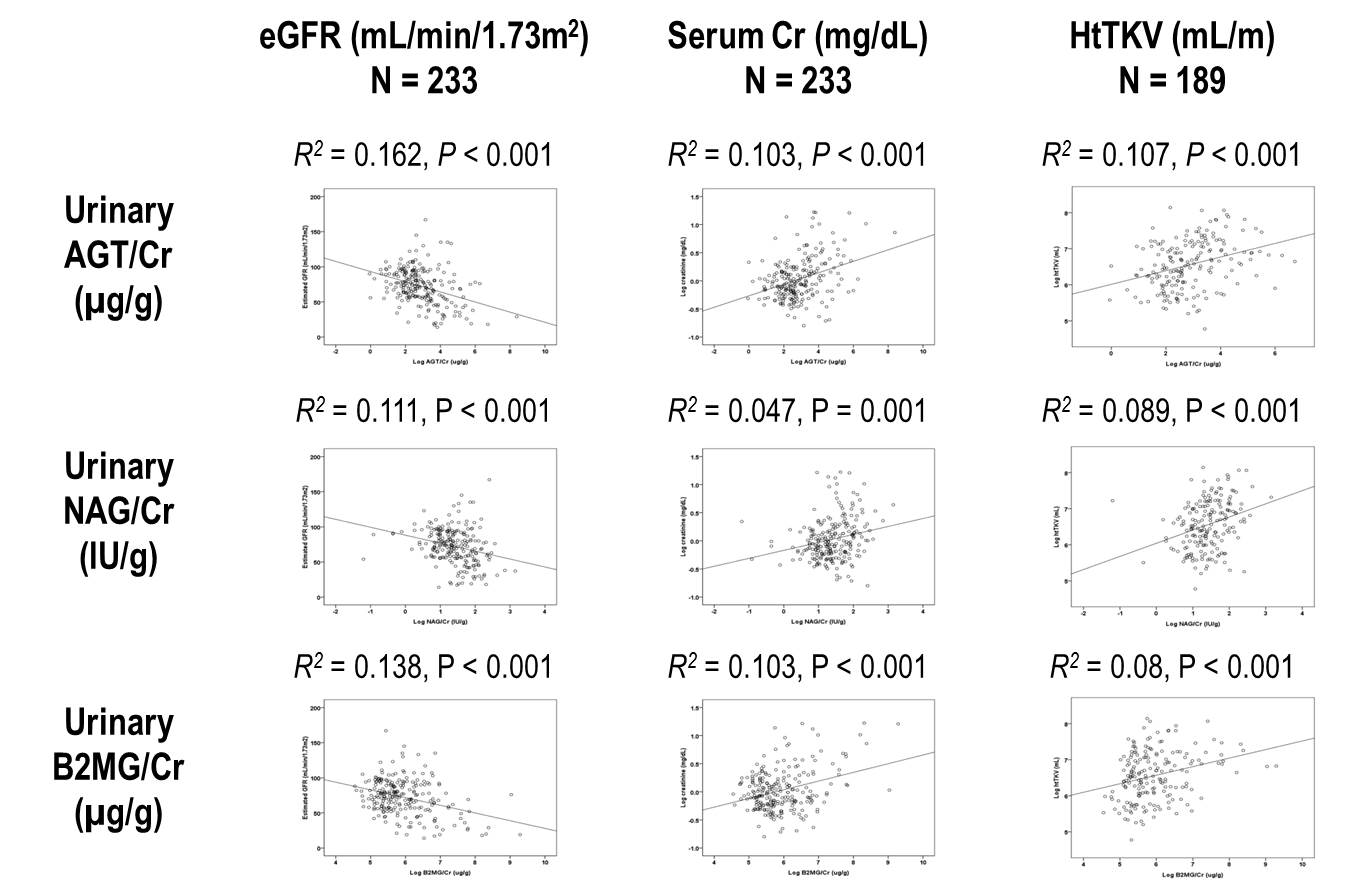


**Supplementary Figure 2. Urinary AGT/Cr, PRA, and Plasma Aldosterone Levels According to RAS Blocker Usage.** Urinary AGT/Cr was not statistically different between patients with RAS blocker usage (n=153, 39.5 ± 70.2 µg/g) and those without RAS blocker usage (n=64, 47.9 ± 117.6 µg/g, *P* = 0.595). In addition, PRA and plasma aldosterone, markers of systemic RAS activation, did not differ between groups (*P* >0.05).


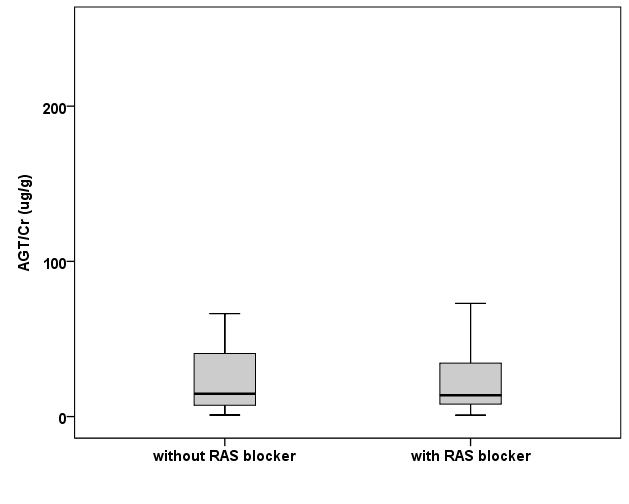

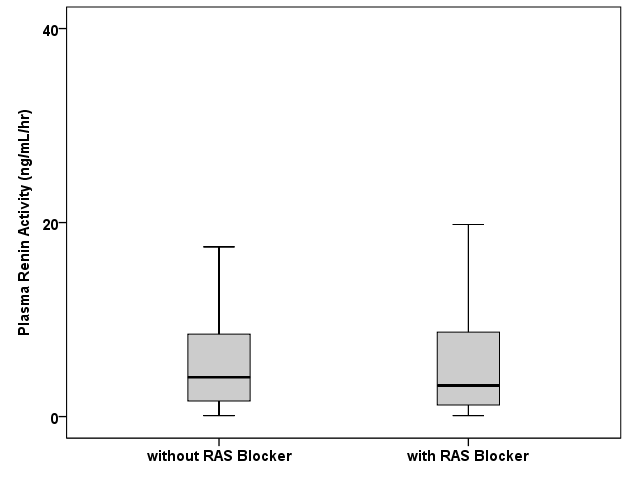

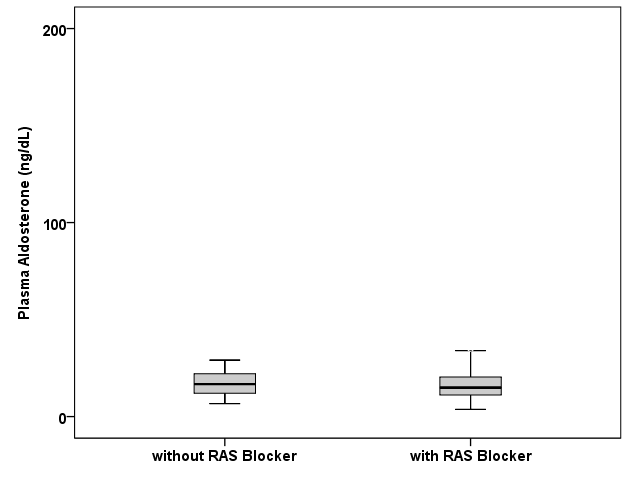


**Supplementary Table 1. Comparison of Urinary AGT/Cr Levels among Different Studies**

| **References** | **Population** | **AGT/Cr (µg/g)** |
| --- | --- | --- |
| Katsurada et al.^1^ | Healthy volunteer (n = 7) | 5.0 – 30.0 |
| Kobori et al.^2^ | Normotensive (n = 36)  Hypertensive w RASB (n = 39)  Hypertensive w/o RASB (n = 31) | 13.7 ± 2.33  13.26 ± 2.6  25.0 ± 4.96 |
| Mills et al.^3^ | Normal control (n = 201, HTN 23.9%)  CKD patients (n = 201, HTN 88.1%) | 4.4 (IQR 3.0, 7.0)  26.3 (5.6, 167.2) |
| Park et al. (current study) | ADPKD (n = 233, HTN 93.1%)   - - CKD stage I-II (n = 186) - - CKD stage IIIA (n = 22) - - CKD stage IIIB (n = 15) - - CKD stage IV-V (n = 9) | 13.7 (7.5, 35.1)  27.8 ± 58.5  56.0 ± 61.1  89.0 ± 89.5  95.3 ± 108.9 |

^1^Katsurada A, Hagiwara Y, Miyashita K, et al. Novel sandwich ELISA for human angiotensinogen. Am J Physiol Renal Physiol 2007; 293(3):F956-60

^2^Kobori H, Alper AB, Jr., Shenava R, et al. Urinary angiotensinogen as a novel biomarker of the intrarenal renin-angiotensin system status in hypertensive patients. Hypertension 2009;53(2):344-50.

^3^Mills KT, Kobori H, Hamm LL, et al. Increased urinary excretion of angiotensinogen is associated with risk of chronic kidney disease. Nephrol Dial Transplant 2012;27(8):3176-81.

ADPKD, autosomal dominant polycystic kidney disease; AGT, angiotensinogen; CKD chronic kidney disease; Cr, creatinine; HTN, hypertension; RASB, renin-angiotensin system blockers.
